# Serum KL-6 and lung ultrasound B-lines: a combined non-invasive model for screening and predicting interstitial lung disease in idiopathic inflammatory myopathy

**DOI:** 10.1136/rmdopen-2026-006708

**Published:** 2026-05-21

**Authors:** Weijin Zhang, Qiongbing Zheng, Shaoyu Zheng, Guangzhou Du, Qichuan Zhang, Shuhan Wu, Guoxin Huang, Zexuan Zhou, Kedi Zheng, Jianqun Lin, Shijian Hu, Qisheng Lin, Jinghua Zhuang, Qingzi Chen, Barbara Ruaro, Anna-Maria Hoffmann-Vold, Marco Matucci-Cerinic, Daniel E Furst, Shaoqi Chen, Yukai Wang

**Affiliations:** 1 Department of Rheumatology and Immunology, Shantou Central Hospital, Shantou, China; 2 Department of Neurology, Shantou Central Hospital, Shantou, China; 3 Department of Radiology, Shantou Central Hospital, Shantou, China; 4 Department of Pulmonary and Critical Care Medicine, Shantou Central Hospital, Shantou, China; 5 Clinical Research Center, Shantou Central Hospital, Shantou, China; 6 Department of Ultrasound, The First Affiliated Hospital of Shantou University Medical College, Shantou, China; 7 Department of Pulmonology, Cattinara Hospital, University of Trieste, Trieste, Italy; 8 Rheumatology Department, Oslo University Hospital, Oslo, Norway; 9 Unit of Immunology, Rheumatology, Allergy and Rare Diseases, IRCCS San Raffaele, Milan, Italy; 10 Vita-Salute San Raffaele University, Milan, Italy; 11 Inflammation Fibrosis and Ageing Initiative (INFLAGE), Division of Genetics and Cell Biology, IRCCS San Raffaele Scientific Institute, Milan, Italy; 12 Division of Rheumatology, Department of Medicine, University of California Los Angeles, Los Angeles, California, USA; 13 University of Washington, Seattle, Washington, USA

**Keywords:** Dermatomyositis, Adult Type, Inflammation, Lung Diseases, Interstitial, Ultrasonography

## Abstract

**Background:**

Interstitial lung disease (ILD) is a major pulmonary complication of idiopathic inflammatory myopathy (IIM), where early diagnosis improves outcomes. While high-resolution CT (HRCT) remains the gold standard, its radiation exposure poses concerns. Serum Krebs von den Lungen-6 (KL-6) and lung ultrasound (LUS) B-lines offer non-invasive alternatives, though their optimal diagnostic cut-offs and combined utility for IIM-ILD remain undefined. This study aimed to establish these cut-offs, evaluate diagnostic performance and develop a clinical prediction model.

**Methods:**

In this single-centre observational study, 162 patients diagnosed with IIM between 2020 and 2024 were enrolled. All underwent serum KL-6 testing, and 120 received systematic 50-point LUS examinations. Using HRCT as the reference standard for ILD diagnosis, receiver operating characteristics (ROC) curve analysis was used to determine optimal cut-offs and construct a combined nomogram.

**Results:**

KL-6 levels were significantly elevated in the ILD group (n=113) compared with non-ILD (n=49). The optimal KL-6 cut-off was 553 U/mL (area under the curve (AUC)=0.895, sensitivity 69%, specificity 95.9%). For LUS B-lines number, 25 was recommended as the clinical threshold (sensitivity 98.8%). Their combination enhanced diagnostic performance (AUC=0.984). An online prediction model demonstrated strong clinical applicability. In an exploratory analysis of the rapid-progressive ILD subgroups, KL-6 and B-lines showed only modest predictive value (AUC ≈ 0.65).

**Conclusion:**

KL-6 ≥553 U/mL and B-lines ≥25 are effective screening thresholds, with combined use significantly improving diagnostic accuracy. The prediction model provides a practical tool for early identification in similar clinical settings. To optimise and generalise its use, external validation in multicentre cohorts is warranted.

**WHAT IS ALREADY KNOWN ON THIS TOPIC**

- Interstitial lung disease (ILD) is a frequent and severe complication of idiopathic inflammatory myopathy (IIM).
- While high-resolution CT is the diagnostic gold standard, its use is limited by radiation exposure.
- Serum Krebs von den Lungen-6 (KL-6) and lung ultrasound B-lines have emerged as promising non-invasive tools, but validated diagnostic thresholds and evidence for their combined use in this specific patient population were lacking.

**WHAT THIS STUDY ADDS**

- This study establishes and validates specific cut-offs for clinical use: KL-6 ≥553 U/mL and B-lines ≥25 as effective screening thresholds for IIM-ILD.
- It demonstrates that the combination of these two non-invasive markers yields superior diagnostic accuracy.
- Furthermore, it provides a practical, web-based prediction model to assist clinicians in early identification and risk stratification.

**HOW THIS STUDY MIGHT AFFECT RESEARCH, PRACTICE OR POLICY**

- The prediction model provides a practical tool for early identification and risk stratification of IIM-ILD, with the potential to optimise clinical decision-making.

## Introduction

 Idiopathic inflammatory myopathy (IIM) is a common group of heterogeneous systemic autoimmune diseases including different subsets. Interstitial lung disease (ILD), as its most frequent and severe pulmonary complication, is a major factor contributing to poor prognosis.^1 2^ Among IIM-ILD cases, rapidly progressive ILD (RP-ILD) represents a clinically critical phenotype characterised by severe disease progression and high mortality, posing significant challenges for clinical management.^3 4^ Notably, a considerable proportion of IIM-patients with ILD lack typical respiratory symptoms in the early stages, often leading to delayed diagnosis and missed optimal treatment windows. Therefore, establishing efficient and reliable early screening strategies is essential for improving patient outcomes and reducing mortality.^5 6^

High-resolution CT (HRCT) is the current imaging gold standard for diagnosing and assessing IIM-ILD and RP-ILD. However, its inherent ionising radiation limits its application in early screening and long-term dynamic follow-up.^7 8^ In recent years, the serum biomarker Krebs von den Lungen-6 (KL-6) and lung ultrasound (LUS) have emerged as two non-invasive, radiation-free detection methods with substantial potential.^9^,^11^ KL-6 is secreted by damaged alveolar type II epithelial cells, and elevated serum levels reflect alveolar epithelial injury and repair processes, closely correlating with ILD activity and severity.^12 13^ LUS enables real-time, visual semiquantitative assessment of pulmonary interstitial lesions through B-line artifacts.^14^

Existing studies suggest a significant correlation between LUS B-lines numbers and KL-6 levels,^15^ and that their combination enhances diagnostic performance for ILD.^1 8^ However, the current evidence primarily consists of retrospective, single-centre studies with limited sample sizes, which challenges the generalisability of these findings and their clinical application. Moreover, the synergistic value of these indicators in predicting RP-ILD risk warrants further investigation through larger and rigorously designed studies.

Building on our clinical practice of routinely combining serum KL-6 and LUS for IIM-ILD screening,^815^,^17^ this study aimed to statistically determine their optimal cut-off values within an IIM population. Furthermore, we sought to develop an intuitive and practical clinical prediction model to aid in screening for ILD and, exploratorily, assessing RP-ILD risk, with the ultimate goal of optimising clinical decision-making.

## Methods

### Study population

This single-centre observational study consecutively enrolled patients diagnosed with IIM at the Department of Rheumatology, Shantou Central Hospital, between January 2020 and December 2024. Diagnosis of IIM strictly adhered to the 2017 European Alliance of Associations for Rheumatology/American College of Rheumatology classification criteria.^18^ All patients underwent chest HRCT. HRCT served as the reference standard for the diagnosis of ILD in this study. To avoid interpretation bias, a senior thoracic radiologist who was blinded to the patients’ clinical data and the results of serum KL-6 and LUS, independently assessed all HRCT scans.^19^ Based on previously reported criteria, with minor modifications, RP-ILD was defined as the occurrence of the following within 1 month of ILD diagnosis: (1) Acute worsening of dyspnoea, (2) Hypoxaemia, defined as an arterial partial pressure of oxygen (PaO₂) <60 mm Hg on room air or a PaO_2_/fraction of inspired oxygen ratio <300 mm Hg, (3) Chest HRCT reveals alveolar exudative lesions (such as ground-glass opacities or consolidation), with or without fibrotic changes (reticular shadows or honeycombing).^20 21^ Exclusion criteria included pregnancy, ILD with other definite causes (eg, occupational dust exposure or drug-related), severe heart failure (New York Heart Association class III-IV), active pulmonary infection and incomplete clinical data.

### Clinical data collection

Clinical data were collected through retrospective analysis of the electronic medical record system, including demographic information (age, sex), disease characteristics (IIM subtype, disease duration from onset, history of malignancy) and serological indicators (myositis-specific antibodies and myositis-associated antibody profiles). Inflammatory markers (including C reactive protein (CRP), erythrocyte sedimentation rate and ferritin) and treatment history (covering the use of glucocorticoids, immunosuppressants and biologics) were also recorded.

### KL-6 measurement

The KL-6 assay was performed using a chemiluminescent enzyme immunoassay kit from Fujirebio (catalogue number: 3FT05TC) on the LUMIPULSE G1200 system. Calibration was conducted with Lumipulse G KL-6 calibrators, with results reported in U/mL. The analytical measuring range was 50–10 000 U/mL, with a lower limit of quantification of 13.47 U/mL. Samples were measured undiluted initially. Those exceeding 10 000 U/mL were diluted at a fixed ratio with Lumipulse G sample diluent 1 and reassayed. Intra-assay and interassay coefficients of variation were 2.4%–3.1% and 3.3%–4.4%, respectively. All samples were analysed in duplicate to ensure reproducibility. It was measured during the initial diagnostic visit at our centre. The median time interval between KL-6 sampling and the HRCT scan was 1 day (IQR 0–3 days).

### LUS examination protocol

LUS examinations were performed by two rheumatologists who underwent unified training and had at least 3 years of operational experience. The operators were blinded to patients’ HRCT results and clinical subgroups. Examinations were conducted using a portable ultrasound device equipped with a convex array probe (frequency 2.5–3.5 MHz). B-lines were defined as discrete laser-like vertical artefacts originating from the pleural line, extending to the bottom of the screen without attenuation, and moving synchronously with lung sliding.^22^ The number of B-lines was recorded at each of 50 scanning points, and the B-lines number was defined as the total number of B-lines across all 50 points.^23^ The intrareader variability was 5.6%, and the inter-reader variability was 7.1%.^1^

### Statistical analysis

Continuous variables were expressed as medians with IQRs and compared using the Mann-Whitney U test. Categorical variables were presented as counts (n) and percentages (%) and compared using the χ^2^ test. Diagnostic accuracy was calculated as (true positives + true negatives) / total cases. Inter-rater reliability was evaluated using Cohen’s κ coefficient. Subsequently, a clinical diagnostic prediction nomogram was developed based on KL-6 levels and the number of LUS B-lines. An interactive web-based version of the nomogram was implemented using shinyapps.io, allowing the prediction probability of IIM-ILD to be calculated and visualised online after entering KL-6 levels and LUS B-lines numbers. Finally, subgroup analyses of RP-ILD were conducted to evaluate the predictive performance of KL-6 levels and LUS B-lines numbers across different patient subgroups. To assess the risk of overfitting, bootstrap internal validation was performed with 1000 resamples using the validate function in the rms package. Model calibration was evaluated using a bootstrap calibration plot, the Hosmer-Lemeshow goodness-of-fit test and the Brier Score. All statistical analyses were conducted using R software (V.4.5.0), and a two-sided value of p<0.05 was considered to indicate statistical significance.

## Results

### Study population characteristics and intergroup comparisons

**Figure 1:**
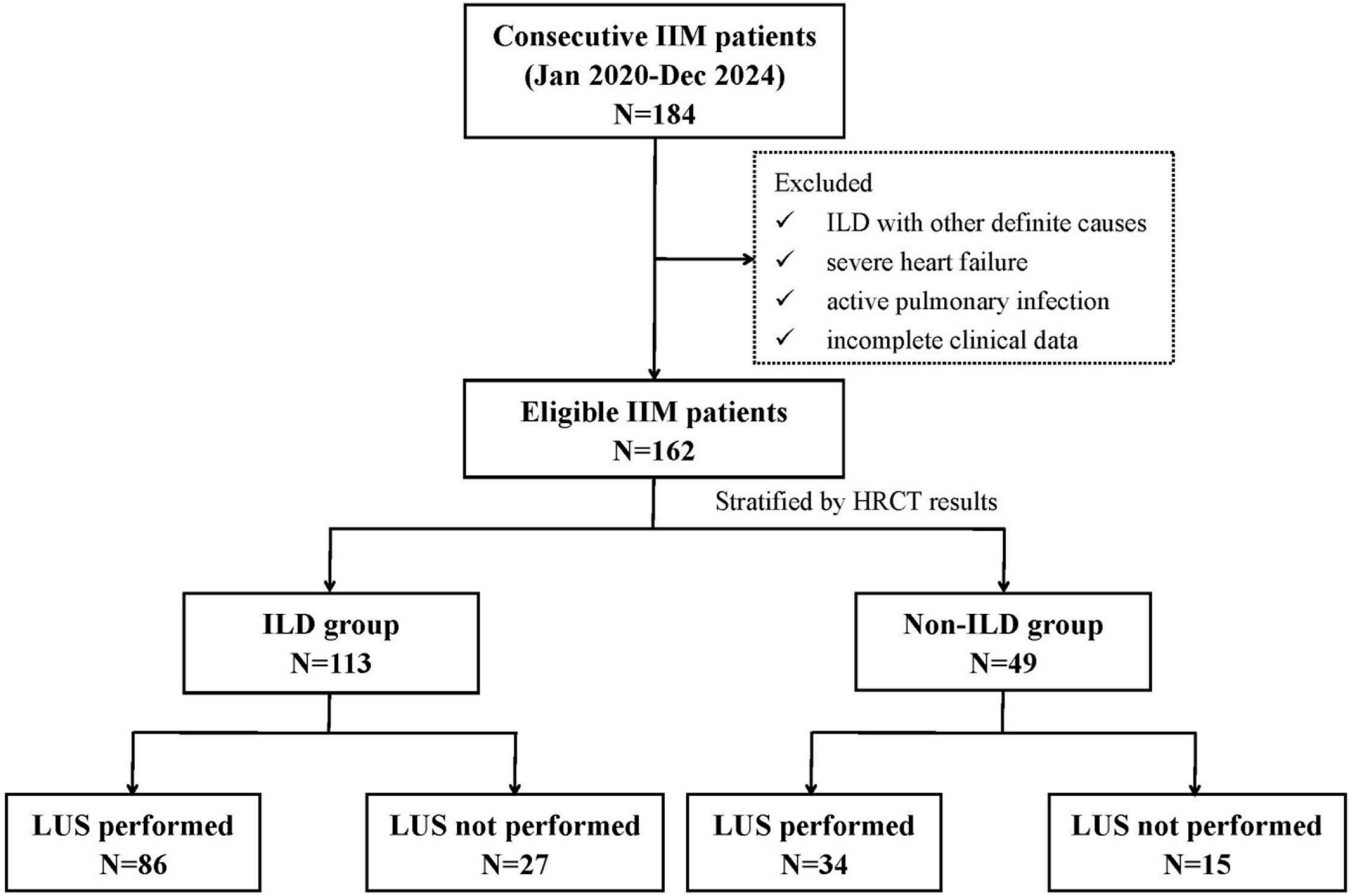
Flow chart of patient enrolment and study groups. HRCT, high-resolution CT; IIM, idiopathic inflammatory myopathy; ILD, interstitial lung disease; LUS, lung ultrasound.

Between January 2020 and December 2024, 184 patients with IIM were consecutively recruited for eligibility screening. After applying the exclusion criteria, 162 patients were ultimately enrolled, constituting the final study cohort (see figure 1 for the patient flow chart). All patients in this cohort completed the HRCT and serum KL-6 measurements. Based on HRCT findings, 113 patients were radiologically diagnosed with ILD, while the remaining 49 comprised the non-ILD group. LUS was completed in 120 patients (74.1%), including 86 (76.1%) from the ILD subgroup and 34 (69.3%) from the non-ILD subgroup. Consequently, this subset of 120 patients with complete data for both KL-6 and LUS was used to develop and analyse the subsequent combined diagnostic model.

In the overall population, women accounted for 72.22% (117 patients), the median age was 56.0 years (IQR 46.2–63.0 years) and the median time from symptom onset to definite diagnosis was 2.00 months (IQR 1.00–6.00 months). Detailed demographic characteristics and laboratory test results are presented in table 1. Intergroup comparisons revealed several significant differences. The ILD group comprised a higher proportion of women, was older and exhibited higher inflammatory markers (such as CRP and erythrocyte sedimentation rate), alongside lower muscle enzyme levels and a lower incidence of malignancy (all p<0.05). The most common cancer types were nasopharyngeal carcinoma (n=7) and lung cancer (n=5), predominantly diagnosed concurrently with IIM; all biomarker assessments were performed prior to initiation of cancer therapy. Regarding myositis-specific antibodies, the ILD group showed significantly higher positivity rates for anti-Jo-1 and anti-MDA5 antibodies, and significantly lower rates for anti-TIF1γ and anti-Mi-2 antibodies (p<0.05). Analysis of medication use indicated that the ILD group had lower usage of methotrexate but significantly higher usage of calcineurin inhibitors, mycophenolate mofetil and cyclophosphamide (p<0.05).

**Table 1:** Demographic, clinical and radiographic data of patients

Baseline characteristics	All patients (n=162)	Patients without radiographic ILD (n=49)	Patients with radiographic ILD (n=113)	P value
Age, years, median (IQR)	56.0 (46.2–63.0)	53.0 (37.0–60.0)	57.0 (49.0–65.0)	0.012
Gender, n (%)				0.003
Female	117 (72.22)	27 (55.10)	90 (79.65)	
Male	45 (27.78)	22 (44.90)	23 (20.35)	
Malignancy, n (%)	18 (11.11)	13 (26.53)	5 (4.42)	<0.001
Disease duration from onset, months, median (IQR)	2.00 (1.00–6.00)	3.00 (1.00–12.00)	2.00 (1.00–6.00)	0.211
CRP, mg/L, median (IQR)	7.02 (3.26–17.62)	3.90 (2.36–10.60)	10.00 (3.64–23.80)	<0.001
CK, U/L, median (IQR)	347.68 (86.50–2006.25)	803.00 (189.00–3350.00)	277.00 (77.00–1635.00)	0.005
Ferritin, ng/mL, median (IQR)	566.60 (313.02–1155.75)	498.05 (249.98–763.25)	656.30 (335.00–1341.25)	0.128
SR, mm/hour, median (IQR)	24.00 (12.00–46.00)	15.00 (8.00–25.00)	30.00 (15.25–48.75)	<0.001
Myositis autoantibodies				
Jo-1, n (%)	31 (19.62)	3 (6.12)	28 (25.69)	0.008
PL7, n (%)	5 (3.21)	0 (0.00)	5 (4.59)	0.323
PL12, n (%)	9 (5.77)	0 (0.00)	9 (8.26)	0.058
EJ, n (%)	9 (5.77)	0 (0.00)	9 (8.26)	0.058
OJ, n (%)	3 (1.92)	0 (0.00)	3 (2.75)	0.554
MDA5, n (%)	39 (25.00)	6 (12.77)	33 (30.28)	0.034
TIF1γ, n (%)	9 (5.77)	9 (19.15)	0 (0.00)	<0.001
NXP2, n (%)	4 (2.56)	3 (6.38)	1 (0.92)	0.082
Mi2, n (%)	4 (2.56)	4 (8.51)	0 (0.00)	0.008
SAE, n (%)	4 (2.56)	2 (4.26)	2 (1.83)	0.584
SRP, n (%)	12 (7.69)	3 (6.38)	9 (8.26)	>0.900
Medications				
GC, n (%)	158 (97.53)	48 (97.96)	110 (97.35)	>0.900
MTX, n (%)	31 (19.14)	21 (42.86)	10 (8.85)	<0.001
AZA, n (%)	1 (0.62)	0 (0.00)	1 (0.88)	>0.900
CNI, n (%)	38 (23.46)	5 (10.20)	33 (29.20)	0.016
JAK, n (%)	21 (12.96)	5 (10.20)	16 (14.16)	0.664
MMF, n (%)	26 (16.05)	3 (6.12)	23 (20.35)	0.042
IVIG, n (%)	36 (22.22)	12 (24.49)	24 (21.24)	0.801
Rituximab, n (%)	1 (0.62)	0 (0.00)	1 (0.88)	>0.900
CTX, n (%)	50 (30.86)	6 (12.24)	44 (38.94)	0.001
KL-6, U/mL, median (IQR)	543.50 (273.50–1097.37)	232.00 (177.39–300.00)	840.79 (477.06–1398.78)	<0.001
B-lines, median, (IQR), n=120	91 (25–208)	12 (10–21)	154 (76–250)	<0.001

AZA, Azathioprine; CK, Creatine kinase; CNI, Calcineurin inhibitor; CRP, C reactive protein; CTX, Cyclophosphamide; EJ, Glycyl-tRNA synthetase; GC, Glucocorticoid; ILD, interstitial lung disease; IVIG, Intravenous immunoglobulin; JAK, Janus kinase inhibitor; Jo-1, Histidyl-tRNA synthetase; KL-6, Krebs von den Lungen-6; MDA5, Melanoma differentiation-associated protein 5; Mi2, Chromodomain-helicase-DNA-binding protein 4; MMF, Mycophenolate mofetil; MTX, Methotrexate; NXP2, Nuclear matrix protein 2; OJ, Isoleucyl-tRNA synthetase; PL7, Threonyl-tRNA synthetase; PL12, Alanyl-tRNA synthetase; SAE, Small ubiquitin-like modifier activating enzyme; SR, Sedimentation rate; SRP, Signal recognition particle; TIF1γ, Transcriptional intermediary factor 1 gamma.

Among the 113 patients with confirmed IIM-ILD, the predominant HRCT pattern was non-specific interstitial pneumonia (NSIP) (n=89, 78.76%). Organising pneumonia (OP) or an NSIP with OP overlap pattern was observed in 62 patients (54.87%), and the usual interstitial pneumonia pattern was found in 20 patients (17.70%).

### Performance of KL-6 in diagnosing IIM-ILD

**Figure 2:**
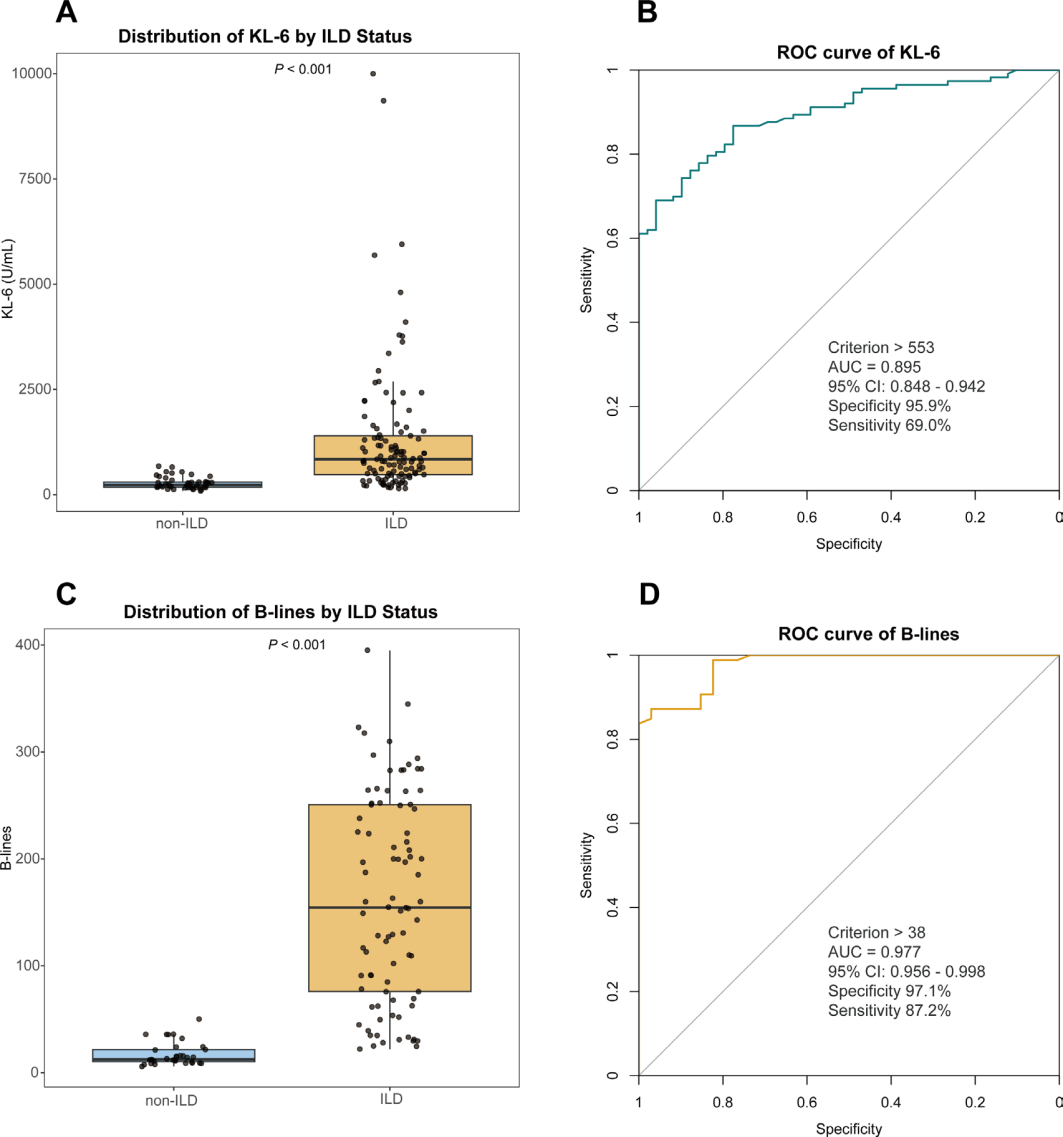
Diagnostic performance of serum KL-6 and LUS in IIM-ILD. (**A**) Comparison of serum KL-6 levels between the ILD and non-ILD groups. (**B**) ROC curve of KL-6 for diagnosing IIM-ILD. (**C**) Comparison of LUS B-lines numbers between the ILD and non-ILD groups. (**D**) ROC curve of B-lines number for diagnosing IIM-ILD. AUC, area under the curve; IIM, idiopathic inflammatory myopathy; ILD, interstitial lung disease; KL-6, Krebs von den Lungen-6; LUS, lung ultrasound; ROC, receiver operating characteristics.

Among the 162 patients, serum KL-6 levels were significantly higher in the ILD group than in the non-ILD group (median 840.79 U/mL, IQR 477.06–1398.78 vs median 232.00 U/mL, IQR 177.39–300.00; p<0.001; figure 2A). Receiver operating characteristics (ROC) curve analysis showed that a KL-6 level exceeding 553 U/mL (area under the curve (AUC) = 0.895, 95% CI 0.848–0.942) was the optimal cut-off value for diagnosing IIM-ILD, with sensitivity and specificity of 69.0% and 95.9%, respectively (figure 2B).

### Performance of LUS in diagnosing IIM-ILD

Among the 162 patients, 120 underwent LUS examination. A comparison of baseline characteristics between patients who did and did not undergo LUS revealed no significant differences (online supplemental table S1). The number of B-lines was significantly higher in the ILD group than in the non-ILD group (median 154, IQR 76–250 vs median 12, IQR 10–21; p<0.001; figure 2C). ROC curve analysis indicated that a B-lines number exceeding 38 (AUC=0.977, 95% CI 0.956 to 0.998) was the optimal cut-off value for diagnosing IIM-ILD, with sensitivity and specificity of 87.2% and 97.1%, respectively (figure 2D). To further evaluate the concordance between LUS and HRCT, we referenced previous studies and set cut-off values of 10 (based on systemic sclerosis^24^) and 12 (based on rheumatoid arthritis research,^25^ which yielded concordance rates of 0.783 (κ value=0.306) and 0.808 (κ value=0.407), respectively. When the cut-off value was set at 25, the concordance rate was highest at 0.942 (κ value=0.850), with a positive predictive value of 0.934, negative predictive value of 0.966, positive likelihood ratio (LR+) of 5.601 and negative likelihood ratio (LR−) of 0.014 (table 2).

**Table 2:** The concordance analysis of LUS and HRCT for IIM-ILD

Threshold	Sensitivity	Specificity	Accuracy	PPV	NPV	LR+	LR−	κ
38	0.872 (0.783–0.934)	0.971 (0.847 to 0.999)	0.900 (0.833–0.942)	0.987 (0.929–1.000)	0.750 (0.597–0.868)	29.651 (4.293–204.818)	0.132 (0.076–0.230)	0.774 (0.002–0.100)
25	0.988 (0.937–1.000)	0.824 (0.655 to 0.932)	0.942 (0.884–0.971)	0.934 (0.862–0.975)	0.966 (0.822–0.999)	5.601 (2.709–11.581)	0.014 (0.002–0.100)	0.850 (0.742–0.957)
12	1.000 (0.958–1.000)	0.324 (0.174 to 0.505)	0.808 (0.729–0.869)	0.789 (0.700–0.861)	1.000 (0.715–1.000)	1.478 (1.172–1.865)	0.000	0.407 (0.231–0.582)
10	1.000 (0.958–1.000)	0.235 (0.107 to 0.412)	0.783 (0.701–0.848)	0.768 (0.679–0.842)	1.000 (0.631–1.000)	1.308 (1.085–1.576)	0.000	0.306 (0.136–0.476)

HRCT, high-resolution CT; IIM, idiopathic inflammatory myopathy; ILD, interstitial lung disease; LR+, positive likelihood ratio; LR−, negative likelihood ratio; LUS, lung ultrasound; NPV, negative predictive value; PPV, positive predictive value.

To quantify the clinical consequences of threshold selection, we performed a burden analysis comparing the two cut-offs (online supplemental table S2). At the threshold of 25 B-lines, 6 of 34 patients without ILD (17.6%) would be referred for unnecessary HRCT, while only 1 of 86 patients with ILD (1.2%) would be missed. At the threshold of 38 B-lines, unnecessary referrals were reduced to 1 patient without ILD (2.9%), but 11 patients with ILD (12.8%) would be missed. Decision curve analysis (DCA) demonstrated that the threshold of 38 B-lines yielded higher net benefit across most probability thresholds, consistent with its superior specificity (online supplemental figure S1).

### Diagnostic performance of combined KL-6 and LUS

**Figure 3:**
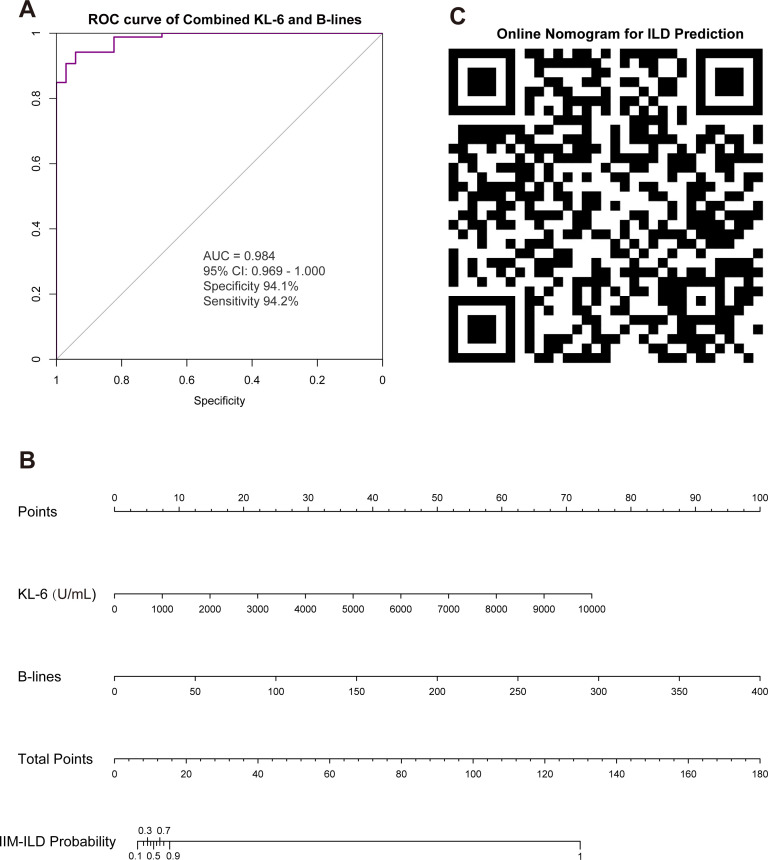
Combined diagnostic performance of KL-6 and LUS B-lines for IIM-ILD and the prediction model. (**A**) ROC curve of the combined KL-6 and LUS B-lines indicators for diagnosing IIM-ILD. (**B**) Nomogram for predicting IIM-ILD probability based on KL-6 level and B-lines number. (**C**) QR code for online access to the nomogram. AUC, area under the curve; IIM, idiopathic inflammatory myopathy; ILD, interstitial lung disease; KL-6, Krebs von den Lungen-6; LUS, lung ultrasound; ROC, receiver operating characteristics.

Among the 120 patients with both KL-6 and LUS B-lines results, combined analysis of these two indicators further increased the AUC to 0.984 (95% CI 0.969 to 1.000), with sensitivity and specificity of 94.2% and 94.1%, respectively (figure 3A). Based on this, we developed a prediction model incorporating KL-6 and B-lines number to simplify clinical application (figure 3B). This prediction nomogram is available online and can be accessed by scanning the QR code (figure 3C). Users need only input the results of these two indicators, and the system will automatically calculate the probability of ILD via an algorithm.

To exclude the potential confounding effects of malignancy and concurrent immunosuppressive therapy, we performed a diagnostic analysis. After excluding 5 patients with lung cancer or 19 patients receiving immunosuppression at the time of initial assessment, the results remained highly consistent with the primary analysis (see online supplemental figures S2 and S3).

As shown in online supplemental table S3, bootstrap internal validation (1000 resamples) demonstrated minimal optimism (0.001), with a corrected AUC of 0.983, confirming the absence of meaningful overfitting. The model showed excellent calibration, with a Hosmer-Lemeshow test value of p=0.999, a mean absolute calibration error of 0.014 and a Brier Score of 0.048 (scaled Brier Score=0.762). The calibration curve demonstrated close agreement between predicted and observed probabilities (online supplemental figure S4). Full regression coefficients and ORs for the combined model are presented in online supplemental table S4.

### Exploratory analysis for RP-ILD prediction

**Figure 4:**
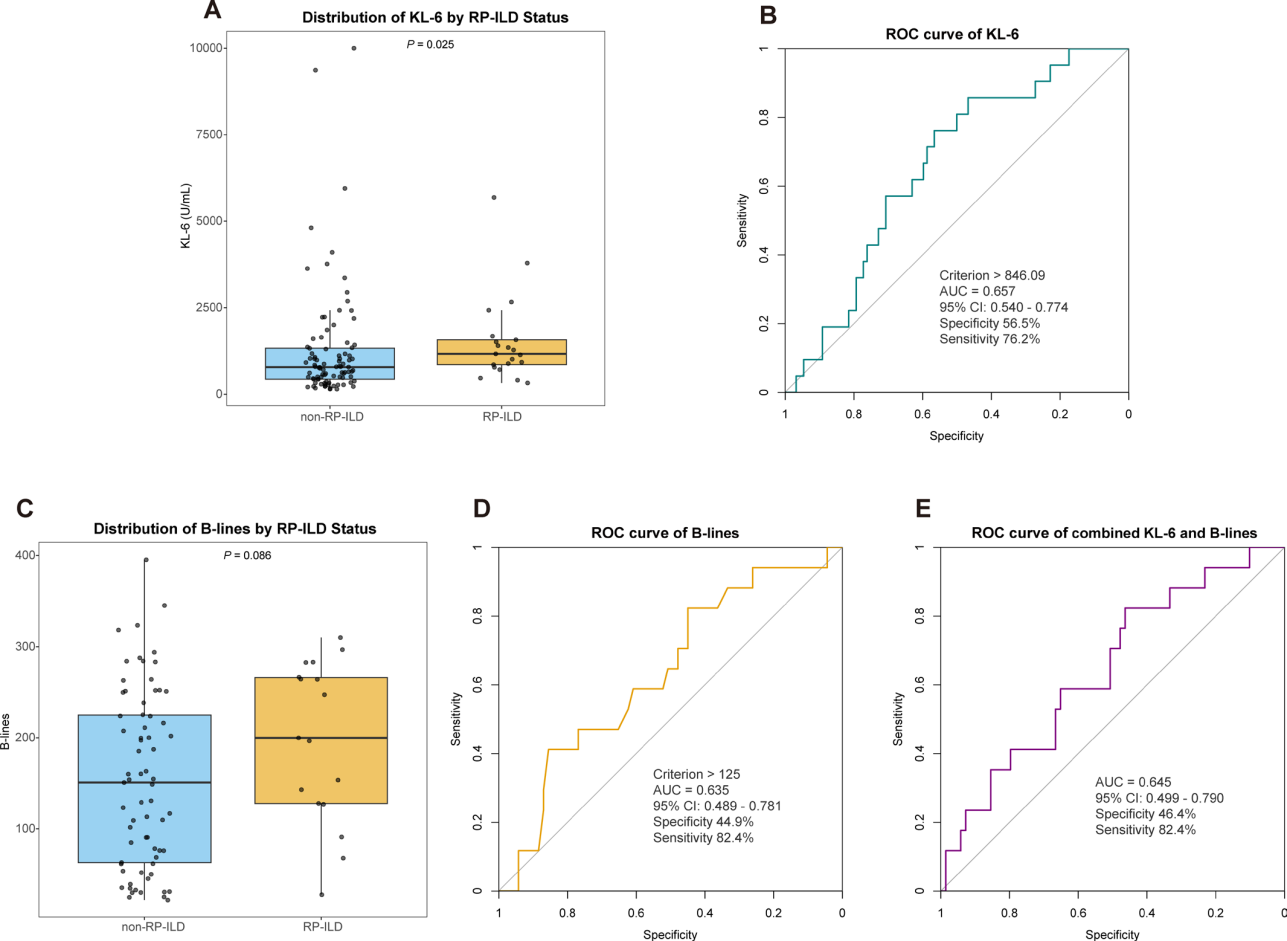
Exploratory analysis of serum KL-6 and LUS in patients with RP-ILD and patients without RP-ILD. (**A**) Comparison of serum KL-6 levels between the RP-ILD and non-RP-ILD groups. (**B**) ROC curve of KL-6 for diagnosing RP-ILD. (**C**) Comparison of LUS B-lines numbers between the RP-ILD and non-RP-ILD groups. (**D**) ROC curve of B-lines number for discriminating RP-ILD. (**E**) ROC curve of the combined KL-6 and LUS B-lines indicators for diagnosing RP-ILD. AUC, area under the curve; KL-6, Krebs von den Lungen-6; LUS, lung ultrasound; ROC, receiver operating characteristics; RP-ILD, rapidly progressive interstitial lung disease.

An exploratory analysis was conducted among the 113 patients with ILD to evaluate biomarkers for predicting RP-ILD, and they were categorised into the RP-ILD group (n=21) and the non-RP-ILD group (n=92) based on disease progression characteristics. As seen in online supplemental table S5, the analysis confirmed that the RP-ILD group had significantly higher rates of oxygen requirement and intensive care unit admission, along with markedly elevated levels of ferritin and CRP. An exploratory analysis revealed that serum KL-6 levels in the RP-ILD group were elevated compared with those in the non-RP-ILD group (median 1163.00 U/mL, IQR 851.39–1569.00; vs median 779.50 U/mL, IQR 436.31–1327.75; p=0.025) (figure 4A). ROC curve analysis for RP-ILD identification showed only modest discriminatory ability, with a KL-6 cut-off level >846.09 U/mL, which yielded a modest AUC of 0.657 (95% CI 0.540 to 0.774), with sensitivity and specificity of 76.2% and 56.5%, respectively (figure 4B). Further analysis of 86 patients with ILD who underwent LUS examination (RP-ILD group: n=17; non-RP-ILD group: n=69) revealed that the LUS B-lines number was higher in the RP-ILD group than in the non-RP-ILD group, though the difference did not reach significance (median 200, IQR 128–266 vs median 151, IQR 63–225; p=0.086; figure 4C). ROC analysis also indicated limited discriminative ability (AUC=0.635, 95% CI 0.489 to 0.781) at a cut-off >125, with sensitivity and specificity of 82.4% and 44.9%, respectively (figure 4D). Among these 86 patients, combined analysis of KL-6 and LUS B-lines metrics did not improve discrimination beyond the single markers, resulting in an AUC of 0.645 (95% CI 0.499 to 0.790) (figure 4E).

## Discussion

Through systematic analysis of clinical data from 162 patients with IIM at our centre, this study confirms the significant value of serum KL-6 and LUS in the diagnosis of IIM-ILD. Our research not only validates the application of these non-invasive tools in IIM-ILD but, more importantly, establishes more precise diagnostic thresholds for this specific population and constructs a practical clinical prediction model.

This study demonstrates that serum KL-6 exhibits excellent diagnostic performance in distinguishing patients with IIM with and without ILD. KL-6 levels were significantly higher in the ILD group than in the non-ILD group, consistent with several previous studies.^1126^,^28^ The optimal diagnostic cut-off value of 553 U/mL (AUC=0.895) determined in our study is close to previous research but offers higher specificity,^12^ providing an optimised threshold for early screening.

Regarding the application of LUS, our study provides important clinical insights. Our team’s previous findings confirmed that LUS B-lines number can effectively assess the disease severity of IIM-ILD through its significant correlation with HRCT fibrosis extent and pulmonary function impairment;^17^ longitudinal monitoring data further demonstrated that dynamic changes in the B-lines number sensitively reflect the response to immunosuppressive therapy in patients with MDA5^+^-dermatomyositis, providing a crucial real-time monitoring tool for efficacy evaluation.^8^ In the present study, we further analysed the cut-off value for LUS in IIM-ILD. Although ROC analysis indicated that B-lines number of 38 had the best diagnostic performance and DCA analysis showed higher net benefit regarding 38 across most probability thresholds, the choice of threshold must be guided by the intended clinical application. It is essential to clarify that our model is positioned as a screening tool, not a diagnostic confirmatory test. For a screening tool designed to minimise missed diagnoses, maximising sensitivity is paramount. Its purpose is to efficiently identify patients with IIM at high risk for ILD who should proceed to definitive HRCT. Therefore, this screening objective directly justifies our recommendation for 25 as B-lines cut-off. While this low threshold achieves excellent sensitivity (98.8%) to avoid missing true cases, we acknowledge the associated clinical consequence: a false-positive rate of approximately 17.6% (specificity 82.4%). In practice, this translates to an increased HRCT referral burden, as roughly one in six screened patients without ILD would be flagged for confirmatory imaging. We argue this is an acceptable and manageable trade-off for a screening stage, where the cost of a false-negative (a missed ILD diagnosis) outweighs that of a false-positive.

The B-lines cut-off value determined in this study is higher than those typically used in rheumatoid arthritis^25^ and systemic sclerosis.^24^ This discrepancy may be attributed to two main reasons: First, the pathophysiology of IIM-ILD, particularly in anti-MDA5 antibody-associated disease, often involves more acute and diffuse pulmonary inflammation, which can generate higher B-lines number. Second, technically, the comprehensive 50-point scanning protocol used in this study, compared with simplified protocols (eg, 14-point protocol) commonly used in studies of other diseases, likely captures more diffuse lesions, resulting in higher absolute counts.

We acknowledge that the 50-point scanning protocol is more time-consuming than simplified schemes (eg, a 14-point protocol). In this study, a comprehensive assessment was necessary to establish a reliable diagnostic model. Future research could focus on extracting the key scanning points from this data set to develop a simplified protocol that maintains diagnostic performance while being more suitable for routine clinical practice.

The combined application of KL-6 and LUS demonstrated significant synergistic effects, elevating the diagnostic performance to an AUC=0.984. The advantage of this multimodal assessment strategy lies in KL-6 providing serological evidence of alveolar epithelial damage,^11 27^ while LUS offers real-time anatomical information on pulmonary interstitial lesions.^7 29^ This complementarity reinforces the multi-indicator combination strategy emphasised in previous studies.^1 16^ Notably, the accompanying online prediction tool provides a practical platform for the integration of these findings, potentially supporting more efficient clinical decision-making.

Compared with comprehensive prediction models for RP-ILD reported in the literature (eg, RP-ILD risk prediction, fever, LDH, age and white cell count models),^3 30 31^ our exploratory analysis, based solely on KL-6 and B-lines, demonstrated only modest predictive performance (AUC ≈ 0.65). This is likely due to the small sample size of RP-ILD-positive cases and the model’s inclusion of only indicators reflecting epithelial injury and interstitial oedema. Previous studies have confirmed that the occurrence of RP-ILD is closely associated with various factors, including myositis-specific antibodies, systemic inflammatory status (eg, elevated CRP, ferritin) and clinical features (eg, fever).^32^,^34^ Therefore, future studies need to expand the sample size and integrate these multidimensional indicators to construct a more accurate prediction system.

The main strengths of this study include the relatively large sample size and the innovative development of a prediction tool. The screening strategy we propose—combined assessment based on the number of LUS B-lines (≥25) and KL-6 (≥553 U/mL)—could represent a practical, non-invasive approach to aid in the early identification of IIM-ILD. However, the study also has several limitations. First, the single-centre design may affect the generalisability of the results. Therefore, external validation in multicentre prospective cohorts is an essential next step. Second, the sample size for the RP-ILD subgroup was limited and requires validation in larger studies. Additionally, as noted in the literature, LUS assessment involves some subjectivity; future research should explore AI-assisted standardised protocols. Moreover, the KL-6 cut-off value established in our study may be overestimated, as the measurements were obtained partly from patients who were not in the early or asymptomatic phases of the disease. Consequently, this could reduce the sensitivity of the biomarker for early screening. Finally, the prediction model included only two core indicators; further studies should incorporate more predictors to improve performance, such as nailfold videocapillaroscopy, which has been associated with lung outcomes in IIM-ILD.^35^

## Conclusion

This study, through systematic evaluation, confirms the significant value of serum KL-6 and LUS B-lines in predicting IIM-ILD and constructs an efficient non-invasive screening strategy. Although further exploration is needed for RP-ILD prediction, the combined prediction model and online tool established in this study provide a promising, non-invasive strategy for the early identification of IIM-ILD, with the potential to improve patient prognosis.

## Supplementary material

10.1136/rmdopen-2026-006708 online supplemental file 1

## Data Availability

Data are available upon reasonable request.
