# Supplementary material for: Serum KL-6 and lung ultrasound B-lines: a combined non-invasive model for screening and predicting interstitial lung disease in idiopathic inflammatory myopathy
Source: RMD Open. 2026 May 21;12(2):e006708. doi: 10.1136/rmdopen-2026-006708 (PMC13202146; doi:10.1136/rmdopen-2026-006708)
Supplement: online supplemental file 1 [file rmdopen-12-2-s001.doc]

**

**

**Figure S1. Decision curve analysis for selecting the optimal LUS B-line cutoff to diagnose IIM-ILD.**

**
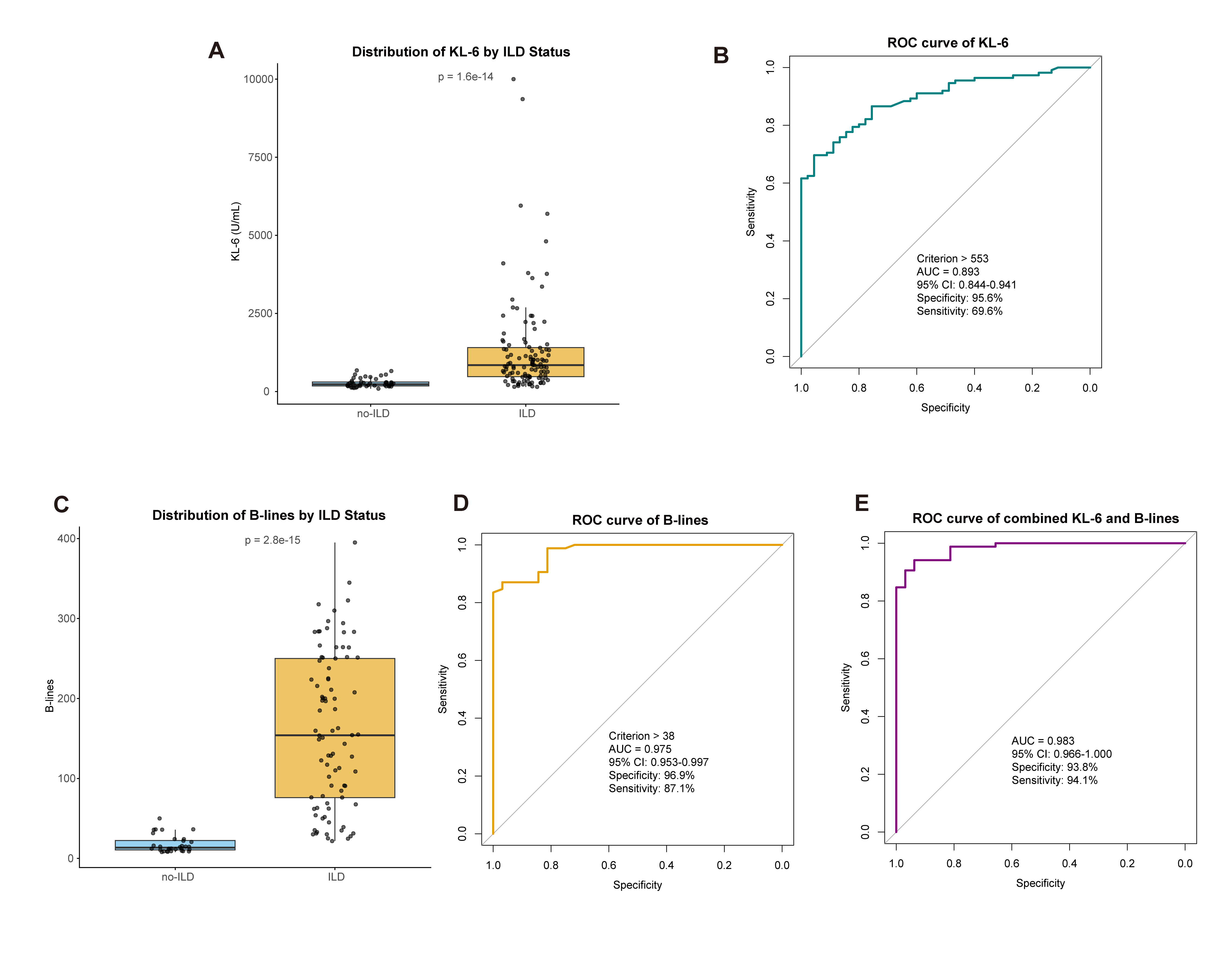
**

**Figure S2. Diagnostic performance of serum KL-6 and LUS in IIM-ILD after excluding 5 patients with lung cancer.**
(A) Comparison of serum KL-6 levels between the ILD and non-ILD groups. (B) ROC curve of KL-6 for diagnosing IIM-ILD. (C) Comparison of LUS B-line counts between the ILD and non-ILD groups. (D) ROC curve of B-line count for diagnosing IIM-ILD. (E) ROC curve of the combined KL-6 and LUS B-line indicators for diagnosing IIM-ILD.

**
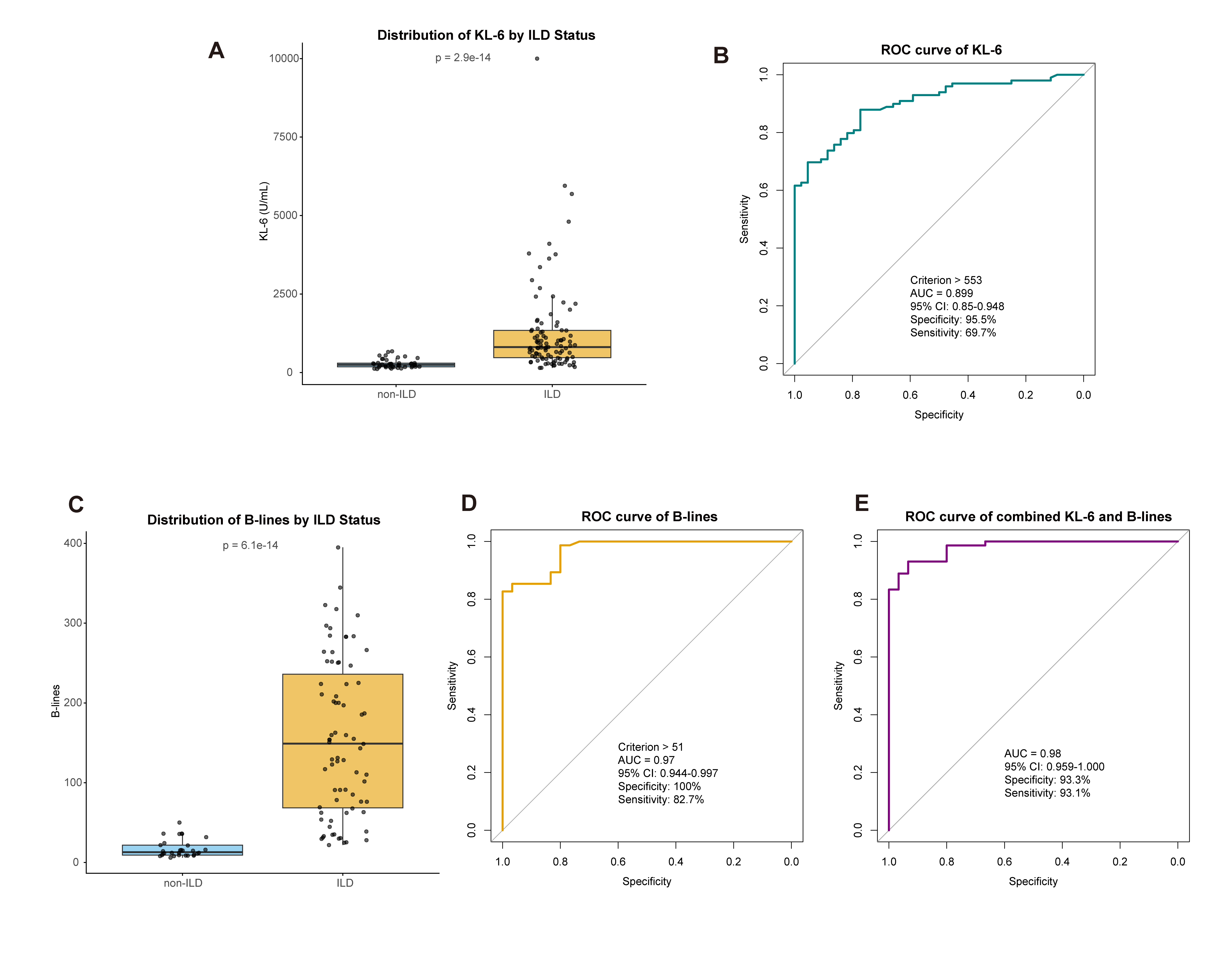
**

**Figure S3. Diagnostic performance of serum KL-6 and LUS in IIM-ILD after excluding 19 patients receiving immunosuppression at the time of initial assessment.**
(A) Comparison of serum KL-6 levels between the ILD and non-ILD groups. (B) ROC curve of KL-6 for diagnosing IIM-ILD. (C) Comparison of LUS B-line counts between the ILD and non-ILD groups. (D) ROC curve of B-line count for diagnosing IIM-ILD. (E) ROC curve of the combined KL-6 and LUS B-line indicators for diagnosing IIM-ILD.

**
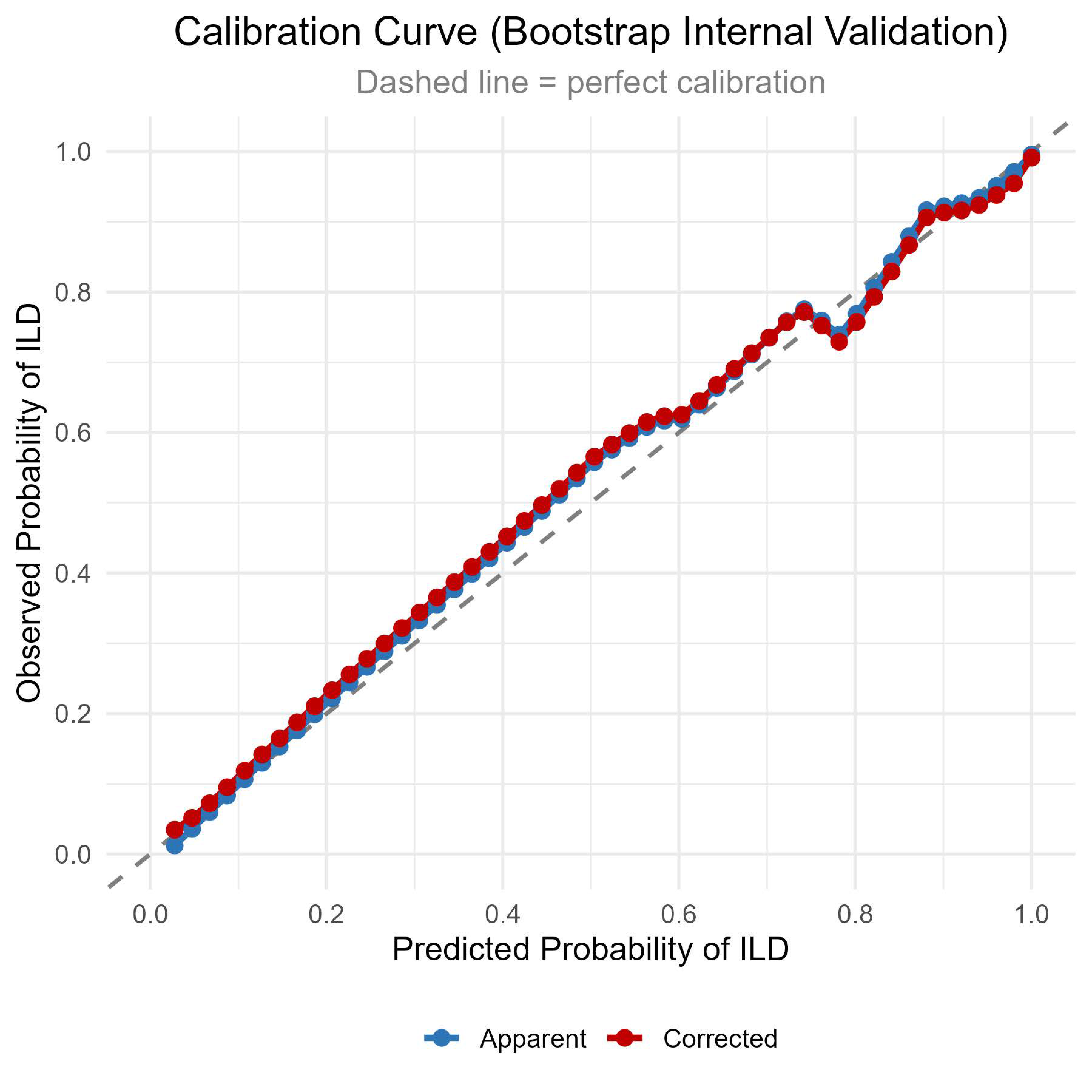
**

**Figure S4. Calibration curve of the diagnostic model.**

**Table S1. Comparison of baseline characteristics between patients with and without LUS performed**

| **Variables** | **All patients (N=162)** | **Patients with LUS performed (N=120)** | **Patients without LUS performed (N=42)** | **p value** |
| --- | --- | --- | --- | --- |
| Age, median (IQR) | 56.00(46.00, 63.00) | 56.00(45.50, 63.00) | 57.00(47.00, 65.00) | 0.758 |
| Gender, n (%) |  |  |  | 0.790 |
| Female | 117(72.22%) | 86(71.67%) | 31(73.81%) |  |
| Male | 45(27.78%) | 34(28.33%) | 11(26.19%) |  |
| Malignancy, n (%) | 18(11.11%) | 13(10.83%) | 5(11.90%) | 0.783 |
| Disease duration, months, median (IQR) | 2.00(1.00, 6.00) | 2.00(1.00, 6.00) | 4.50(1.00, 12.17) | 0.124 |
| CRP, median (IQR) | 7.02(3.26, 17.69) | 7.52(3.28, 16.95) | 6.96(2.93, 23.80) | 0.823 |
| CK, median (IQR) | 347.69(86.00, 2009.00) | 333.00(83.50, 2003.50) | 479.00(108.00, 2438.00) | 0.644 |
| Ferritin, median (IQR) | 566.60(308.70, 1163.00) | 624.20(315.45, 1198.50) | 470.30(308.70, 1113.00) | 0.868 |
| ESR, median (IQR) | 24.00(12.00, 46.00) | 24.00(12.00, 46.00) | 22.00(9.00, 46.00) | 0.800 |
| ILD, n (%) | 113 (69.75%) | 86 (71.67%) | 27(64.29%) | 0.370 |
| KL-6, median (IQR) | 543.50(273.00, 1108.00) | 583.50(271.50, 1086.75) | 460.00(275.00, 1171.60) | 0.570 |
| Myositis autoantibodies |  |  |  |  |
| Jo-1, n (%) | 31(19.62%) | 23(19.33%) | 8(20.51%) | 0.872 |
| PL-7, n (%) | 5(3.21%) | 4(3.39%) | 1(2.63%) | >0.900 |
| PL-12, n (%) | 9(5.77%) | 6(5.08%) | 3(7.89%) | 0.689 |
| EJ, n (%) | 9(5.77%) | 7(5.93%) | 2(5.26%) | >0.900 |
| OJ, n (%) | 3(1.92%) | 2(1.69%) | 1(2.63%) | 0.570 |
| MDA5, n (%) | 39(25.00%) | 32(27.12%) | 7(18.42%) | 0.282 |
| TIF1γ, n (%) | 9(5.77%) | 7(5.93%) | 2(5.26%) | >0.900 |
| NXP2, n (%) | 4(2.56%) | 3(2.54%) | 1(2.63%) | >0.900 |
| Mi-2, n (%) | 4(2.56%) | 3(2.54%) | 1(2.63%) | >0.900 |
| SAE, n (%) | 4(2.56%) | 3(2.54%) | 1(2.63%) | >0.900 |
| SRP, n (%) | 12(7.69%) | 10(8.47%) | 2(5.26%) | 0.732 |

**Table S2.** Quantitative comparison of HRCT referral burden between two LUS B-line thresholds (n = 120)

|  | **Cutoff ≥25 B-lines** | **Cutoff ≥38 B-lines** |
| --- | --- | --- |
| True positives (ILD correctly identified) | 85 | 75 |
| False negatives (ILD missed) | 1 (1.2%) | 11 (12.8%) |
| False positives (unnecessary HRCT referrals) | 6 (17.6%) | 1 (2.9%) |
| True negatives | 28 | 33 |
| Sensitivity | 98.8% | 87.2% |
| Specificity | 82.4% | 97.1% |
| False discovery rate | 6.6% | 1.3% |
| FP per TP detected | 0.071 | 0.013 |

**Table S3. Summary of bootstrap internal validation and calibration metrics for the combined KL-6 and B-line model.**

| **Metric** | **Value** |
| --- | --- |
| Apparent AUC | 0.984 |
| Bootstrap-corrected AUC | 0.983 |
| Optimism | 0.001 |
| Mean absolute calibration error | 0.014 |
| Brier Score | 0.048 |
| Scaled Brier Score | 0.762 |
| Hosmer-Lemeshow χ² | 0.592 |
| Hosmer-Lemeshow p-value | 0.999 |

**Table S4. Logistic regression coefficients, odds ratios, and 95% confidence intervals for the combined KL-6 and B-line prediction model.**

| **Term** | **Coefficient** | **OR** | **95% CI** | **P value** |
| --- | --- | --- | --- | --- |
| Intercept | −5.356 | 0.005 | 0.000 – 0.042 | <0.001 |
| KL-6 | 0.004 | 1.004 | 1.000 – 1.008 | 0.056 |
| B-lines | 0.123 | 1.131 | 1.066 – 1.237 | 0.001 |

**Table S5. Comparison of clinical characteristics between non-RP-ILD and RP-ILD patients**

| **Variables** | **Non-RP-ILD (N=92)** | **RP-ILD (N=21)** | **p value** |
| --- | --- | --- | --- |
| LDH, median (IQR) | 411.00 (312.60, 605.75) | 412.20 (303.00, 456.00) | 0.398 |
| CRP, median (IQR) | 7.50 (3.30, 16.65) | 47.00 (12.90, 72.40) | <0.001 |
| ESR, median (IQR) | 28.00 (14.00, 46.00) | 48.50 (26.25, 63.25) | 0.054 |
| Ferritin, median (IQR) | 532.80 (282.50, 1218.00) | 1105.00 (484.15, 1551.50) | 0.036 |
| Oxygen requirement, n (%) | 42 (45.65%) | 21 (100.00%) | <0.001 |
| ICU admission, n (%) | 3 (3.26%) | 13 (61.90%) | <0.001 |
